# Effects of Nanoplastics on the Dinoflagellate *Amphidinium carterae* Hulburt from the Perspectives of Algal Growth, Oxidative Stress and Hemolysin Production

**DOI:** 10.3390/nano11102471

**Published:** 2021-09-22

**Authors:** Su-Chun Wang, Fei-Fei Liu, Tian-Yuan Huang, Jin-Lin Fan, Zhi-Yin Gao, Guang-Zhou Liu

**Affiliations:** 1 Institute of Marine Science and Technology, Shandong University, Qingdao 266237, China; suchunw@163.com (S.-C.W.); huangty1994@163.com (T.-Y.H.); gzy18864805967@163.com (Z.-Y.G.); 2 Department of Science and Technology Management, Shandong University, Jinan 250100, China; fanjinlin@sdu.edu.cn

**Keywords:** polystyrene nanoplastics, growth inhibition, oxidative stress, hemolytic toxin

## Abstract

Recently, the effects of nanoplastics (NPs) on aquatic organisms have attracted much attention; however, research on the toxicity of NPs to microalgae has been insufficient. In the present study, the effects of polystyrene nanoplastics (nano-PS, 50 nm) on growth inhibition, chlorophyll content, oxidative stress, and algal toxin production of the marine toxigenic dinoflagellate *Amphidinium carterae* Hulburt were investigated. Chlorophyll synthesis was promoted by nano-PS on day 2 but was inhibited on day 4; high concentrations of nano-PS (≥50 mg/L) significantly inhibited the growth of *A. carterae*. Moreover, despite the combined effect of superoxide dismutase (SOD) and glutathione (GSH), high reactive oxygen species (ROS) level and malondialdehyde (MDA) content were still induced by nano-PS (≥50 mg/L), indicating severe lipid peroxidation. In addition, the contents of extracellular and intracellular hemolytic toxins in nano-PS groups were significantly higher than those in control groups on days 2 and 8, except that those of extracellular hemolytic toxins in the 100 mg/L nano-PS group decreased on day 8 because of severe adsorption of hemolytic toxins to the nano-PS. Hence, the effects of nano-PS on *A. carterae* are closely linked to nano-PS concentration and surface properties and exposure time. These findings provide a deep understanding of the complex effects of NPs on toxigenic microalgae and present valuable data for assessing their environmental risks.

## 1. Introduction

Plastic pollution in aquatic environments has recently gained attention worldwide. With the in-depth study of microplastics (MPs), nanoplastics (NPs) have also drawn public awareness [1]. Nanoplastics, defined as plastic materials with at least one dimension on the nanoscale, have been found in natural waters and their concentrations are expected to continuously increase because of the degradation of primary micro(nano)plastics [2,3,4,5]. Owing to their small size and large surface area, the ecological effects of NPs on aquatic organisms differ from those of large plastic particles [6]. Polystyrene (PS; 100 nm, <10 mg/L) has been found to accumulate in crustaceans without affecting their mortality rate; however, the swimming speed and enzyme activity in individuals with high accumulated PS levels are significantly altered [7]. When exposed to PS-COOH (100 nm), a dose-dependent relationship between reactive oxygen species (ROS) production and PS-COOH concentration was observed in the sperm cells of Pacific oysters [8]. In addition, PS-NH_2_ NPs interfere with the development of sea urchin embryos by modulating protein and gene profiles [9]. Overall, NPs pollution in aquatic environments has become a main challenge that requires further investigation.

As the basis of the food web, algae are crucial for the stability of marine ecosystems [10]. Owing to their short growth period and high sensitivity to toxic substances, microalgae have been considered a good choice for detecting environmental threats caused by MPs pollution [11]. Size-dependent negative effects of PS with the particle sizes of 0.05, 0.5, and 6 μm on the marine flagellate *Dunaliella tertiolecta* have been reported [12]. In addition to growth inhibition effects, polystyrene nanoplastics (nano-PS) can also reduce chlorophyll content and accelerate ROS production in algal cells [13]. For example, after 2 d of exposure to PS-NH_2_ (200 nm), the chlorophyll content and photosynthetic efficiency of *Chaetoceros neogracile* decreased by 24% and 13%, respectively, and esterase activity also significantly decreased while the intracellular ROS level increased [14]. In addition, MPs and NPs can also affect the secretion of hemolytic toxins produced by harmful algal bloom species and absorb some substances of hemolytic toxins released by algal cells [15,16,17,18,19]. Considering the coexistence of NPs and harmful algal bloom species in aquatic environments, interactions between NPs and hemolytic toxins may negatively affect aquatic ecology and pose a potential risk to animals and humans. Therefore, more attention should be paid to the comprehensive evaluation of the effects of NPs on harmful algal bloom species.

*Amphidinium carterae* Hulburt (*A. carterae*), a harmful algal bloom species, is mainly distributed in tropical and temperate seas worldwide and can produce hemolytic toxins [20,21,22]. The synthesis of hemolytic toxins has been reported to be closely related to the salinity, pH, temperature, and light intensity of the algal growth environment [23,24,25,26]. *A. carterae* cells in the logarithmic phase have been shown to increase their hemolytic activity as light intensity increased, while hemolytic activity was greatly inhibited at low temperature (10 °C) and salinity (15) [26]. In addition, current research on the effects of NPs on the toxin production of harmful algal bloom species is still insufficient and unclear; for example, PS (100 nm) has no significant effect on the growth and photosynthetic activity of *Microcystis aeruginosa*, and promotes microcystin production only after 48 h. PS-NH_2_ (50 nm) induces photosynthesis inhibition and oxidative stress, and enhances the synthesis of microcystin; while larger particle PS-NH_2_ (200 nm, 5 mg/L) had no significant effect on microcystin production of *M. aeruginosa* [18,19,27]. Thus, a deeper understanding of the effects of NPs on harmful algal bloom species needs to be investigated.

In order to evaluate the effects of NPs on marine harmful algal bloom species, we chose nano-PS (50 nm) without functional groups as the test chemical and *A. carterae* as the test species. We investigated the growth inhibition, chlorophyll content, ROS level, antioxidant enzyme activity, and hemolytic toxin content of *A. carterae*. In addition, scanning electron microscopy (SEM) was performed to observe the interaction between nano-PS and algal cells. This research forms the basis for a more comprehensive evaluation of the toxicity of NPs to marine harmful algal bloom species and for the assessment of their environmental risks.

## 2. Materials and Methods

### 2.1. Algal Cultivation

*A. carterae* was provided by Shanghai Guangyu Biotechnology Co., Ltd. (Shanghai, China). Microalgae were cultivated in f/2 medium made with sterile artificial seawater (filtered through a 0.45 μm acetate filter membrane). Erlenmeyer flasks containing microalgal cells were cultivated at 20 ± 1 °C under cool white fluorescent lights (4000 lux) with a 12 h-light-dark cycle and were kept at a constant temperature oscillation incubator (ZQZY-CGF8, Zhichu Instrument Co., Ltd., Shanghai, China) at a speed of 50 rpm. According to the growth curves of *A. carterae* (Figure S1), the incubation lasted approximately 4 days until logarithmic phase growth prevailed. Cell density was calculated using an optical microscope (BX53, Olympus, Tokyo, Japan).

### 2.2. Nanoplastics Treatment

Nano-PS powder with a size of 50 nm was purchased from Changchun Lianyu Chemical Technology Co., Ltd. (Changchun, China) (Figure S2). The hydrodynamic diameter and zeta potential of nano-PS were measured using a particle size analyzer (Zetasizer Nano ZS90, Malvern Panalytical Ltd., Malvern, UK), which were 424 nm and −23.7 mV, respectively. Before treatment, the nano-PS and culture media (10 mL) were added to Erlenmeyer flasks and ultrasonicated to obtain a uniformly dispersed suspension. Then, 40 mL of algal cells in logarithmic phase growth were transferred into the flasks. The initial algal density was maintained at 9 × 10^5^ cells/mL, while the concentrations of nano-PS were set at 10, 20, 50, 80, and 100 mg/L. Each concentration treatment was replicated three times, and all operations were performed under sterile conditions to avoid contamination by bacteria. All flasks were placed in an incubator for 8 days under pre-culture conditions.

### 2.3. Measurement of Chlorophyll Content

Chlorophyll content was determined using the acetone extraction method [28]. First, 20 mL of algal culture was centrifuged (5000 rpm, 10 min) to remove the supernatant. The pellets were mixed with 5 mL of 90% acetone to extract chlorophyll for 24 h at 4 °C in the dark. The samples were then centrifuged at 6500 rpm for 15 min, and the absorbance (OD) of the supernatant at 645 nm and 663 nm was measured. Total chlorophyll (*T*_chl_) content was calculated using the following equation: *T*_chl_ = 20.2OD_645_ + 8.02OD_663_.

### 2.4. Assays for ROS Level, MDA Content, SOD Activity, and GSH Content

In this study, ROS levels were detected using 2′, 7′-dichlorodihydrofluorescein diacetate (DCFH-DA; Sigma-Aldrich, St. Louis, MO, USA), based on previous studies [29,30]. The stock solution of DCFH-DA was prepared using N, N-dimethylformamide (DFM, Sinopharm Chemical Reagent Co., Ltd., Shanghai, China) and its final concentration used in the experiment was 10 μmol/L. The microalgal cells immersed in DCFH-DA solution were incubated in the dark at 37 °C for 20 min. Finally, these algal cells were resuspended in PBS and detected using a multi-mode microplate reader (Synergy H1, BioTek, Winooski, VT, USA) to obtain the fluorescence intensity values, which indicated the ROS level.

The SOD activity, MDA content, and GSH content were measured using detection kits purchased from the Jiancheng Bioengineering Institute (Nanjing, China). First, algal cells were crushed using an ultrasonic cell disruptor. After adding the relevant reagents, samples for SOD detection were incubated at 37 °C for 20 min. The samples for MDA were kept in a water bath at 95 °C for 40 min, and for GSH, at room temperature for 5 min before measurement. Then, SOD, MDA, and GSH were determined using the Synergy H1 system at 450, 532, and 420 nm, respectively.

### 2.5. Extraction and Determination of Hemolytic Toxin

In this experiment, the hemolytic toxin content was measured using an erythrocyte lysis assay (ELA) [31,32]. First, 0.4 mL of different concentrations of digitalis saponin and 1.6 mL of 0.5% rabbit red blood cells were incubated in a 37 °C water bath for 30 min. The reaction liquid was then centrifuged, and the absorbance of the supernatant was measured at 540 nm to obtain the hemolysis standard curve of digitalis saponin (Figure S3). The extracellular toxin of *A. carterae* was determined from the supernatant after centrifugation (5000 rpm, 10 min), and the remaining algal cells were used to determine the content of intracellular toxins. For the intracellular toxin extraction, algal cells were first crushed using an ultrasonic cell disruptor; then, an extraction solution prepared using chloroform, methanol, and water (13:7:5, *V:V:V*) was added for liquid phase extraction. After evaporation with a rotary evaporator, intracellular toxins were collected using 1 mL of methanol. The above ELA test was repeated to obtain the corresponding hemolytic toxin content based on the hemolysis standard curve [33,34].

### 2.6. Sample Preparation for SEM Assay

The morphology of the algal cells was observed using a scanning electron microscope (FEI Quanta 250 FEG; Thermo-Fisher Scientific, Waltham, MA, USA). Microalgal cells were collected by centrifugation (5000 rpm, 10 min) and fixed with 2.5% glutaraldehyde at 4 °C overnight. Then, the samples were washed three times with phosphate buffer (PBS, pH 7.4) and dehydrated using 30, 50, 70, 80, 90, 95 and 100% ethanol solutions for 15 min, respectively [27,35]. Finally, the samples were freeze-dried for SEM observation.

### 2.7. Statistical Analysis

All tests were performed in triplicate, and the results were expressed as the mean ± standard deviation. The enzyme analysis results were analyzed using one-way ANOVA and LSDs, with the analysis performed using the SPSS statistical software (IBM, Chicago, IL, USA). A value of *p* < 0.05 was used to denote a significant difference.

## 3. Results and Discussion

### 3.1. Cell Density and Chlorophyll Content

**Figure 1:**
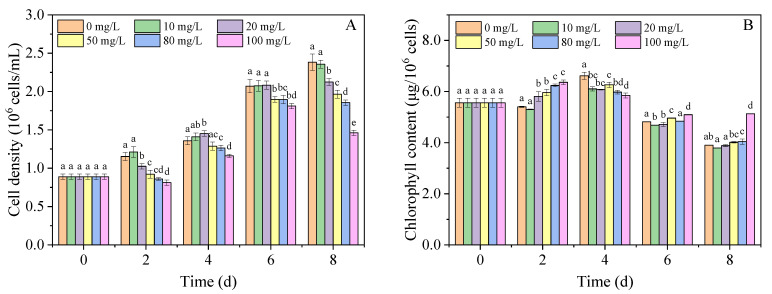
Cell density (**A**) and chlorophyll content (**B**) of *A. carterae* at different concentrations of nano-PS. Different letters represent significant differences (*p* < 0.05).

As shown in Figure 1A, the effects of nano-PS on the cell density of *A. carterae* were related to its concentration and experimental time. The nano-PS at 10 mg/L had no significant effect on the growth of algal cells, whereas 20 mg/L of nano-PS inhibited the growth of algal cells only on days 2 and 8 compared with the control group. The nano-PS at 50, 80, and 100 mg/L significantly inhibited the growth of the test algal cells with the *IR* of 17.5, 22.1, and 38.7% (Table S1), respectively. There was a clear negative dose-dependent relationship between cell density and nano-PS concentration on day 8. In addition, 100 mg/L of nano-PS induced the algal cells to enter the decline phase earlier than the other treatments. Moreover, we also observed that the *IR* of nano-PS groups, except that of the nano-PS at 10 mg/L, decreased initially and then increased (Table S1). This may have resulted from the resistance and adaptation of algae to nano-PS [36].

The chlorophyll content of *A. carterae* increased first and then decreased during the experiment (Figure 1B), owing to the limited nutrients with increasing cell density. On day 2, nano-PS at 20–100 mg/L promoted the synthesis of chlorophyll; the chlorophyll content in the 100 mg/L nano-PS group was 1.18-fold higher than that in the control group (Table S2). This may be a stress response to the decrease in light intensity caused by nano-PS [37,38]. As the cell density decreased in the nano-PS groups, pigment accumulated in the algal cells on 6–8 d, resulting in much higher chlorophyll content in the 100 mg/L nano-PS group than in the control group. However, nano-PS decreased the chlorophyll content on day 4, probably because of ROS accumulation, which could inhibit the synthesis of chlorophyll [39].

### 3.2. Lipid Peroxidation in A. carterae

**Figure 2:**
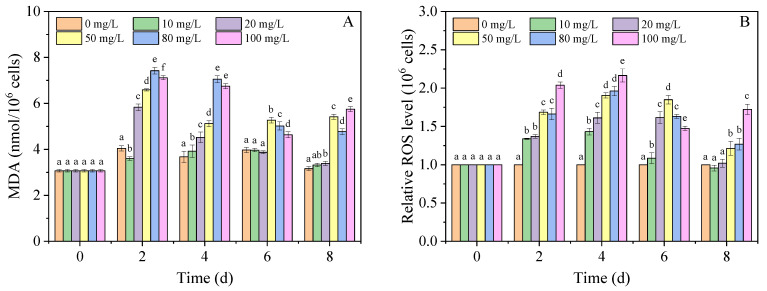
The MDA content (**A**) and relative ROS level (**B**) of *A. carterae* in different concentrations of nano-PS groups. Different letters represent significant differences (*p* < 0.05).

Malonaldehyde is the main peroxidation product of cytomembrane lipids and is often caused by excessive ROS [40,41]. In the present study, the MDA content and ROS levels were measured, and the results are shown in Figure 2A,B. Compared with the control group, the nano-PS (≥20 mg/L) significantly increased the MDA content on days 2 and 4, indicating severe lipid peroxidation caused by nano-PS. In addition, low concentrations (≤20 mg/L) of nano-PS had no significant effect on MDA content of *A. carterae* on 6 and 8 d. However, the MDA content in the 50, 80, and 100 mg/L nano-PS groups was also significantly higher than that in the control group, but their difference gradually decreased, probably because of the adaptability of algal cells to the stress of nano-PS [42]. These results were confirmed by the relative ROS levels shown in Figure 2B. The relative ROS levels in the nano-PS groups were much higher than those in the control group on 2 and 4 d, and nano-PS at high concentrations (≥50 mg/L) induced high ROS levels over the experimental period. Similarly, Hazeem et al. reported that nano-PS (20 and 50 nm) could cause a significant increase in ROS levels in *Chlorella vulgaris* [43]. Overall, the high ROS level and MDA content indicate the occurrence of lipid peroxidation, which probably induces membrane damage.

### 3.3. SOD Activity and GSH Content

**Figure 3:**
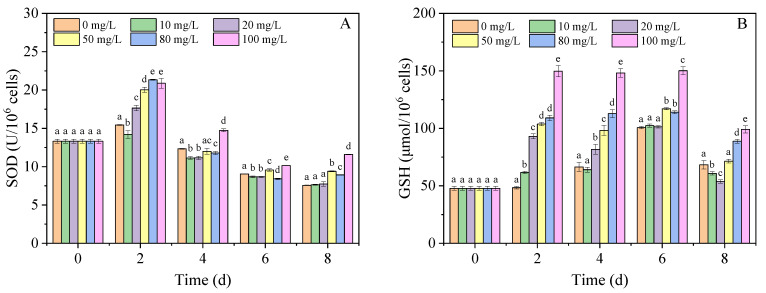
The SOD activity (**A**) and GSH content (**B**) of *A. carterae* in different concentrations of nano-PS groups. Different letters represent significant differences (*p* < 0.05).

As important antioxidants, both SOD and GSH can remove ROS in cells to protect cells against oxidative damage [41,44]. To explore the oxidative stress response caused by nano-PS in the algal cells, the SOD activity and GSH content were detected, and the results are displayed in Figure 3A and Figure 3B, respectively. The SOD activity in the nano-PS groups, except the 10 mg/L nano-PS group, was significantly higher than that in the control group on day 2. The SOD activity in all groups gradually decreased, especially from 2 d to 4 d. The rate of decrease was 18.97% in the control group, and 40.6, 43.1, and 29.7 in 50, 80 and 100 mg/L of nano-PS groups, respectively. These changes are most likely because of the inhibition of SOD synthesis caused by the high ROS levels in the test algae cells [45]. In addition, the gap in SOD activity between the nano-PS groups and the control was obviously reduced, thus indicating that the antioxidant capacity of algal cells decreased [46]. Despite the decrease in SOD activity, 100 mg/L of nano-PS stimulated SOD activity at all times.

Similarly, the GSH content of *A. carterae* increased initially and then decreased over the experimental period (Figure 3B). The GSH content in nano-PS groups was significantly higher than that in the control group on days 2–4. Moreover, there was a dose-dependent relationship between the content of GSH and the concentrations of nano-PS; nano-PS at 100 mg/L induced the highest GSH content, which was up to 3.1- and 2.2-fold higher than that of the control group, respectively. In contrast to SOD activity, the GSH content in the high concentration nano-PS groups was still significantly higher than that in the control group from 4 d to 8 d. This can compensate for the decrease in SOD activity caused by nano-PS at high concentrations to reduce ROS levels. The complex variation trend of the SOD activity and GSH content may be caused by the content of antioxidant enzymes induced by nano-PS and the complementary effect between these enzymes [44,47,48]. Overall, although nano-PS induced high ROS levels, the algal cells still had an antioxidant capacity to resist lipid peroxidation throughout the experimental period [42,49].

### 3.4. SEM Analysis

**Figure 4:**
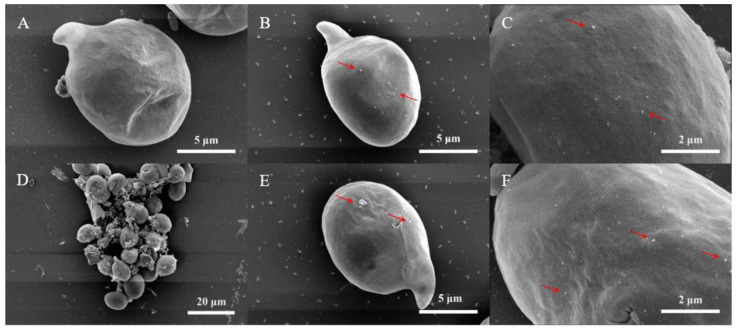
SEM images of *A. carterae* in the control (**A**), 100 mg/L of nano-PS (**B**–**D**) on day 2, and 100 mg/L of nano-PS (**E**,**F**) on day 8. The arrows point to the nano-PS adhered to the algae cell.

As shown in Figure 4, although nano-PS induced membrane oxidative damage, it had no visible effect on size and morphology of the test algal cells compared with those in the control group because of the protection by the cell wall. However, the nano-PS at high concentrations aggregated easily and adsorbed on the surface of the algal cells (Figure 4B,C,E,F). Additionally, nano-PS and algal cells can even form large heterogeneous aggregates (Figure 4D) which sink easily; this aggregation is mainly caused by extracellular polymeric substances [50,51,52]. In all, the adsorption and aggregation of nano-PS with microalgae could limit the transfer of energy and nutrients, and the motility of the algal cells, thus inhibiting microalgal growth [53].

### 3.5. Hemolytic Toxins Content

**Figure 5:**
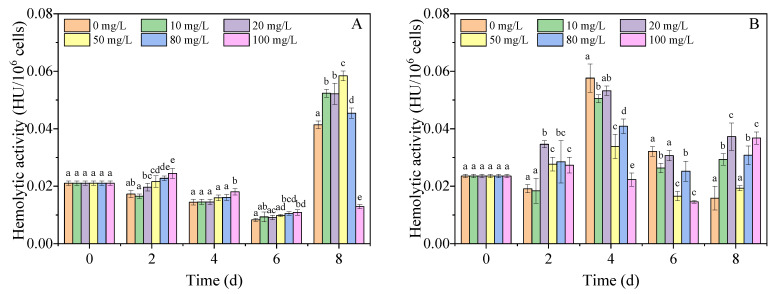
Hemolytic activity of extracellular toxins (**A**) and intracellular toxins (**B**) in different concentrations of nano-PS groups. Different letters represent significant differences (*p* < 0.05).

In the present study, the hemolytic activities of extracellular and intracellular toxins were assessed to evaluate the effect of nano-PS on toxin production. Based on the results shown in Figure 5, the contents of extracellular and intracellular hemolytic toxins were higher than those of the control group under the stimulation of nano-PS on day 2. This can be attributed to the stress reaction of algae in adverse environments, in which the oxidative stress of nano-PS enhances the expression of toxic genes [18,23,54,55]. From 4 d to 6 d, the content of extracellular toxin in the nano-PS groups was not significantly different from that in the control group, while the content of intracellular hemolytic toxin in the nano-PS groups decreased, most likely because of the growth inhibition caused by nano-PS. On the last day, the content of intracellular hemolytic toxin in the nano-PS groups increased again. In addition, owing to the cell membrane damage caused by the high ROS level, the content of extracellular hemolytic toxin in the nano-PS groups (10–80 mg/L) also increased [19]. However, the content of extracellular hemolytic toxin in the 100 mg/L nano-PS group was significantly lower than that in the control group because some substances in hemolytic toxins, such as amphidinols, could be adsorbed by nano-PS [15,16]. Based on the above results, a high concentration of nano-PS can affect not only the synthesis of toxins but also the concentration of toxins in algal cells.

## 4. Conclusions

In the present study, the effects of nano-PS (50 nm) on the toxigenic dinoflagellate *A. carterae* were investigated in terms of algal growth, oxidative stress, and hemolysin production. The results showed that the effects of nano-PS on algal cells depended greatly on its concentration and exposure time. Nano-PS significantly inhibited chlorophyll synthesis only on day 4. Moreover, nano-PS at high concentrations inhibited cell growth at all time, while it stimulated first then limited cell growth at low concentrations. Although the antioxidant capacity of algal cells was higher than that in control group, nano-PS especially at high concentrations could still induce high ROS levels and lipid peroxidation, which were the main cause of cell growth inhibition. In addition, nano-PS can affect not only the synthesis of toxins, but also the toxin distribution in and out of algal cells. Considering the coexistence of NPs and harmful algal bloom species, their interaction may have negative consequences for aquatic ecology, thus further affecting the aquaculture industry and posing a potential risk to animals and humans. These findings of this research are valuable for us to understand the effects of NPs on harmful algal bloom species and provide insights into assessing their actual risks to the environment.

## Data Availability

The datasets generated during and/or analyzed during the current study are available from the corresponding author on reasonable request.

## Supplementary Materials

The following are available online at https://www.mdpi.com/article/10.3390/nano11102471/s1, Figure S1: Growth curve of *A. carterae*, Figure S2: TEM image of nano-PS, Figure S3: The hemolysis standard curve of digitalis saponin. The value of EC_50_ is 1.53 μg/mL, which means 1.53 μg/mL digitalis saponins is equal to 1 HU, Table S1: Growth inhibition rate (*IR*) of nano-PS on *A. carterae*, Table S2: Inhibition rate of nano-PS on chlorophyll content of *A. carterae*.
