# Supplementary material for: Effects of Nanoplastics on the Dinoflagellate Amphidinium carterae Hulburt from the Perspectives of Algal Growth, Oxidative Stress and Hemolysin Production
Source: Nanomaterials (Basel). 2021 Sep 22;11(10):2471. doi: 10.3390/nano11102471 (PMC8541305; doi:10.3390/nano11102471)
Supplement: Supplementary file 1 [file nanomaterials-11-02471-s001.zip › nanomaterials-1370686-supplementary.pdf]

Supplementary data for

Effects of Nanoplastics on the Dinoflagellate *Amphidinium carterae* Hulburt from the Perspectives of Algal Growth, Oxidative Stress and Hemolysin Production

Su-chun Wang [1], Fei-fei Liu [1,*], Tian-yuan Huang [1], Jin-Lin Fan [2], Zhi-yin Gao [1] and Guang-zhou Liu [1,*]

[1] Institute of Marine Science and Technology, Shandong University, Qingdao 266237, PR China; suchunw@163.com (S.-C.W.); huangty1994@163.com (T.-Y.H.); gzy18864805967@163.com (Z.-Y.G.)

[2] Department of Science and Technology Management, Shandong University, Jinan 250100, China; fanjinlin@sdu.edu.cn (J.-L.F)

* Correspondence: liufeifei@sdu.edu.cn (F.-F.L.); liuguangzhou@sdu.edu.cn (G.-Z.L.); Tel.: +86 532 5863 3262 (F.-F.L.)

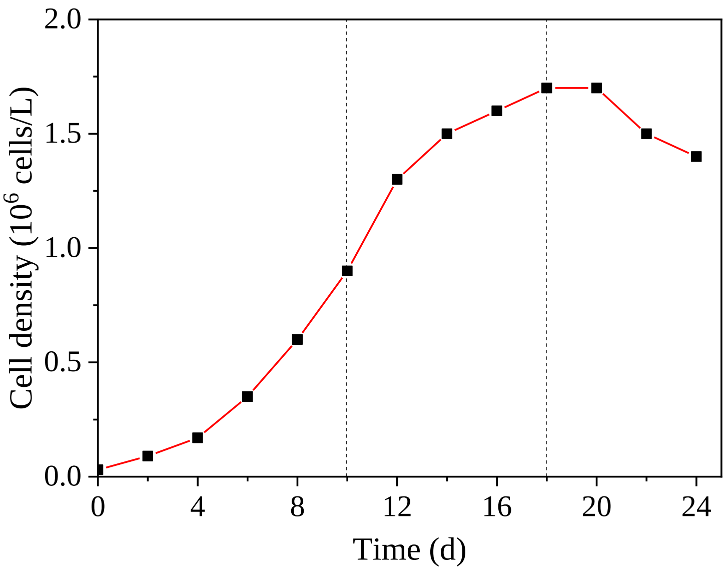

Figure S1. Growth curve of *A. carterae*.

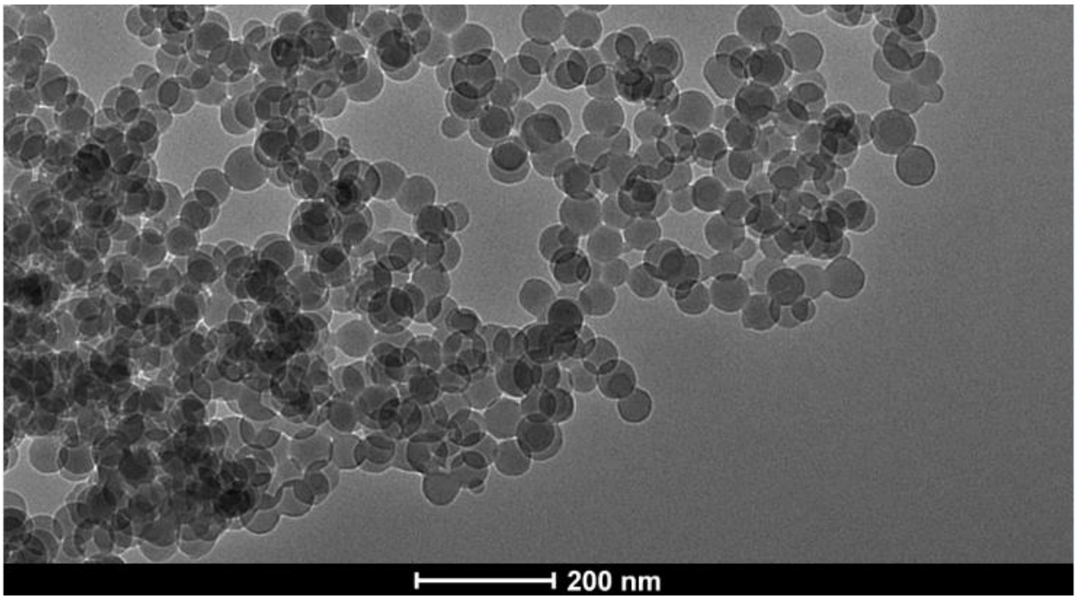

Figure S2. TEM image of nano-PS.

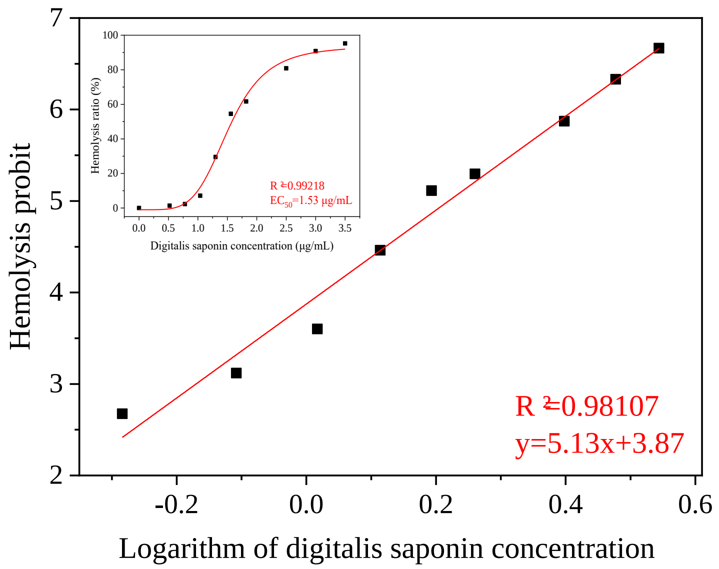

Figure S3. The hemolysis standard curve of digitalis saponin. The value of EC_{50} is 1.53 μg/mL, which means 1.53 μg/mL digitalis saponins is equal to 1 HU.

Table S1. Growth inhibition rate (*IR*) of nano-PS on *A. carterae*.

Time (d)	*IR* (%)				
	10 mg/L	20 mg/L	50 mg/L	80 mg/L	100 mg/L
2	−5.0%	11.1%	20.0%	25.4%	29.6%
4	−3.8%	−6.9%	5.1%	6.9%	14.4%
6	−0.2%	0.6%	8.4%	8.5%	13.1%
8	1.1%	10.8%	17.5%	22.1%	38.7%

The *IR* was calculated using the following equation: *IR* (%) = $(C_0 - C_i)/C_0$, where C_0 and C_i are the cell densities of the control and experimental groups.

Table S2. Inhibition rate of nano-PS on chlorophyll content of *A. carterae*.

Time (d)	Nano-PS Concentration (mg/L)				
	10	20	50	80	100
2	−3.57%	6.28%	12.75%	14.11%	15.99%
4	3.68%	0.53%	7.80%	19.81%	16.94%
6	3.14%	3.19%	6.65%	9.07%	9.29%
8	7.76%	14.39%	18.33%	21.36%	20.98%

The calculation method of inhibition rate of chlorophyll content refers to the growth inhibition rate (Table S1).
